# Influenza A virus replication has a stronger dependency on Raf/MEK/ERK signaling pathway activity than SARS-CoV-2

**DOI:** 10.3389/fcimb.2023.1264983

**Published:** 2023-10-26

**Authors:** Helen Hoffmann, Marina Ebensperger, Annika Schönsiegel, Hazem Hamza, Julia Koch-Heier, André Schreiber, Stephan Ludwig, Michael Schindler, Oliver Planz

**Affiliations:** ^1^ Department of Immunology, Interfaculty Institute for Cell Biology, Eberhard Karls Universitaet Tuebingen, Tuebingen, Germany; ^2^ Atriva Therapeutics GmbH, Tuebingen, Germany; ^3^ Virology Laboratory, Environmental Research Division, National Research Centre, Giza, Egypt; ^4^ Institute of Virology (IVM), Westfaelische Wilhelms Universitaet, Muenster, Muenster, Germany; ^5^ Department of Molecular Virology, Institute for Medical Virology and Epidemiology of Viral Disease, University Hospital Tuebingen, Tuebingen, Germany

**Keywords:** SARS-CoV-2, influenza virus, EC_50_, IC_50_, Raf/MEK/ERK, MEK inhibitor, antiviral, pandemic preparedness

## Abstract

The recent COVID-19 pandemic again highlighted the urgent need for broad-spectrum antivirals, both for therapeutic use in acute viral infection and for pandemic preparedness in general. The targeting of host cell factors hijacked by viruses during their replication cycle presents one possible strategy for development of broad-spectrum antivirals. By inhibiting the Raf/MEK/ERK signaling pathway, a central kinase cascade of eukaryotic cells, which is being exploited by numerous viruses of different virus phyla, the small-molecule MEK inhibitor zapnometinib has the potential to address this need. We here performed a side-by-side comparison of the antiviral efficacy of zapnometinib against IAV and SARS-CoV-2 to determine the concentration leading to 50% of its effect on the virus (EC_50_) and the concentration leading to 50% reduction of ERK phosphorylation (IC_50_) in a comparable manner, using the same experimental conditions. Our results show that the EC_50_ value and IC_50_ value of zapnometinib are indeed lower for IAV compared to SARS-CoV-2 using one representative strain for each. The results suggest that IAV’s replication has a stronger dependency on an active Raf/MEK/ERK pathway and, thus, that IAV is more susceptible to treatment with zapnometinib than SARS-CoV-2. With zapnometinib’s favorable outcome in a recent phase II clinical trial in hospitalized COVID-19 patients, the present results are even more promising for an upcoming phase II clinical trial in severe influenza virus infection.

## 1 Introduction

As of today, virus infections, especially those with pandemic potential, still pose a major threat to humans as broad-spectrum antiviral therapies to treat acute infection are still missing. Present approaches to control virus infection are highly specific and mainly include preventive measures such as vaccination or treatment of acute infections with direct-acting antivirals. Both approaches are compromised by the fast appearance of new virus variants escaping the preventive and therapeutic measures. The coronavirus disease 2019 (COVID-19) pandemic has clearly demonstrated the need for broad-spectrum antiviral drugs that are effective in treating more than just one specific virus variant, virus species, or phylum. One strategy to develop long-lasting broad-spectrum antiviral drugs includes targeting host cell proteins that are used by a broad number of viruses to enable their replication (Schor and Einav, 2018; Lesch et al., 2019; Gutierrez-Chamorro et al., 2021; Meineke et al., 2022; Wild et al., 2022). An advantage of this strategy is that host cell targets are not affected by the fast mutation rate of the virus and are therefore expected not to be prone to resistance development. On the other hand, host cell targeting drugs need to be carefully assessed for potential toxic side effects on the host. The rapid accelerated fibrosarcoma/mitogen-activated protein kinase kinase/extracellular signal-regulated kinase (Raf/MEK/ERK) signaling pathway represents one of those targets for broad-spectrum antiviral drug development as it is exploited by many viruses during their replication cycle, e.g., coronaviruses, Ebola virus, hepatitis C virus, respiratory syncytial virus, yellow fever virus, Borna disease virus, and influenza virus (Planz et al., 2001; Pleschka et al., 2001; Zampieri et al., 2007; Pleschka, 2008; Menzel et al., 2012; Albarnaz et al., 2014; Bonjardim, 2017; Preugschas et al., 2019; Schreiber et al., 2020; Meineke et al., 2022; Schreiber et al., 2022). Zapnometinib, a small-molecule inhibitor, targets MEK1/2, the central kinase of the Raf/MEK/ERK pathway, and thereby blocks its downstream activities. Zapnometinib has been developed for the treatment of severe respiratory viral infections with complications of hyperinflammation. The antiviral activity of zapnometinib treatment has been demonstrated for influenza virus infection *in vivo* (Laure et al., 2020; Koch-Heier et al., 2022) and influenza virus and severe acute respiratory syndrome coronavirus 2 (SARS-CoV-2) infection *in vitro* (Laure et al., 2020; Schreiber et al., 2020; Schreiber et al., 2022). Furthermore, zapnometinib has been shown to be well tolerated in a phase I clinical trial (ClinicalTrials.gov NCT04385420) in healthy volunteers and is considered safe to be used in humans.

Influenza viruses are enveloped, negative-sense, single-stranded RNA viruses that belong to the family of *Orthomyxoviridae* with influenza A, B, and C virus causing infection in human. Until today, influenza A virus (IAV) poses a great threat to human health having caused several pandemics with high mortality, e.g., the Spanish flu in 1918, the Asian flu in 1957, and the swine flu pandemic in 2009 (Taubenberger and Morens, 2006; Dawood et al., 2012; Viboud et al., 2016). IAV infects epithelial cells by binding with its hemagglutinin (HA) spikes to sialic acid residues on the host cell surface (Couceiro et al., 1993). For successful fusion of the virus envelope with the host cell membrane, the HA precursor protein needs to be cleaved by serine proteases into HA1 and HA2 during replication of the virus. Host cell proteases facilitating this step are, among others, the transmembrane protease, serine 2 (TMPRSS2), the transmembrane protease, serine 4 (TMPRSS4), and the human airway trypsin-like protease (HAT) (Steinhauer, 1999; Böttcher-Friebertshäuser et al., 2010). Following attachment, the virus enters the cell via endocytosis. Unlike most other RNA viruses, replication of influenza viruses takes place in the nucleus (Herz et al., 1981). Later, virus budding and release of new virions occur at the cell membrane (reviewed in Nayak et al., 2004). For IAV, activation of the RAF/MEK/ERK signaling pathway has been shown to occur in an unusual biphasic manner, with a transient activation early in the replication cycle within the first 30 min of infection and a second, more sustained activation phase at 8–10 h post-infection (Pleschka et al., 2001).

SARS-CoV-2, an enveloped, positive-sense single-stranded RNA virus belonging to the family of *Coronaviridae*, has caused the most recent pandemic with more than 700 million confirmed cases of COVID-19, leading to more than 6 million deaths worldwide (WHO Coronavirus (COVID-19) Dashboard). SARS-CoV-2 can enter host cells by binding to receptors like the angiotensin-converting enzyme 2 (ACE2). For efficient fusion and entry, the SARS-CoV-2 spike protein needs to be cleaved by suitable host cell proteases on the cell surface like TMPRSS2. Another entry route is via endocytosis where endosomal cathepsins can cleave the spike protein to facilitate fusion (Hoffmann et al., 2020). Unlike IAV, SARS-CoV-2 replicates solely in the cytoplasm. Assembly and budding of new virions take place at the endoplasmic reticulum Golgi intermediate compartment (ERGIC) (Scherer et al., 2022). Activation of the Raf/MEK/ERK signaling pathway has been shown to be an early and transient event in the replication cycle and was detected within the first 2 h post-infection in Calu-3 cells (Schreiber et al., 2022). Furthermore, Forsyth et al. found that binding of Spike S1 to the ACE2 receptor leads to activation of the Raf/MEK/ERK signaling pathway and the induction of cytokines (Forsyth et al., 2022). *In vivo*, the activation of the signaling pathway has been shown in response to infection in a COVID-19 mouse model. MEK inhibition could circumvent this effect (Xie et al., 2022).

**Figure 1:**
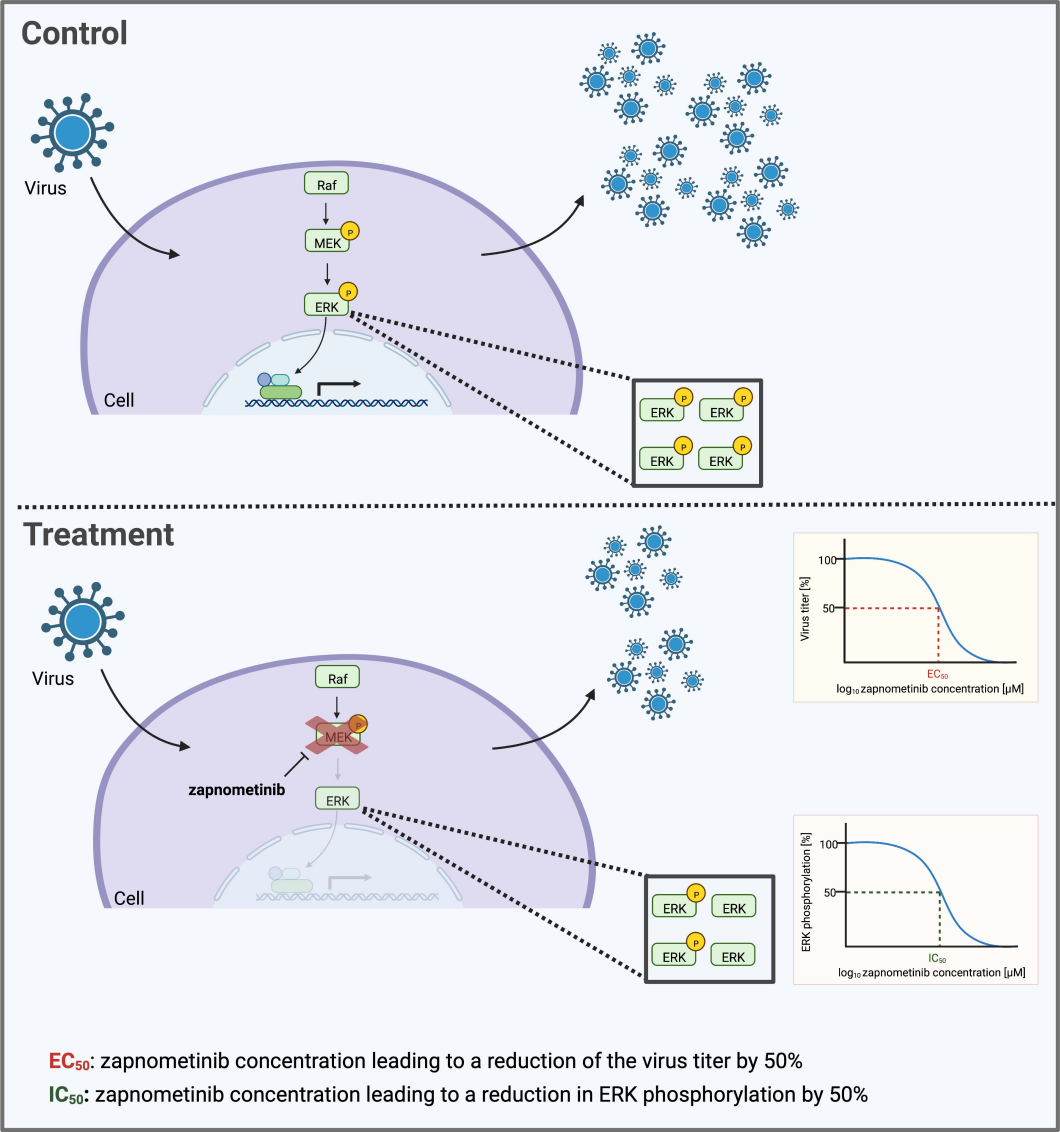
Schematic depicting the determination of the IC_50_ and EC_50_ value of the MEK inhibitor zapnometinib. Many viruses depend on an active Raf/MEK/ERK signaling pathway in their host cell to facilitate their replication. Treatment with zapnometinib inhibits the kinase MEK1/2 and thereby prevents phosphorylation of its substrate ERK1/2. Reduced ERK phosphorylation in turn decreases downstream activities of the pathway including the support of virus replication. The inhibition of ERK phosphorylation and virus replication follows a dose-dependent course. Key figures characterizing the efficacy of a drug are the EC_50_ and IC_50_ value that can be calculated based on the dose–response curve. This graph was created with BioRender.com.

With IAV and SARS-CoV-2 both being dependent on active MEK1/2 to sustain their replication, we were interested in a direct comparison of the drug efficacy of zapnometinib. Key figures used to characterize a drug’s efficacy are the IC_50_ and EC_50_ concentration. The IC_50_ value represents the concentration of an inhibitor leading to 50% inhibition of its target and the EC_50_ the concentration at which 50% of the drug’s effect is obtained. In the case of zapnometinib, the IC_50_ is the concentration at which 50% of MEK1/2 inhibition is observed. With ERK1/2 being the main substrate of MEK1/2, MEK inhibition is routinely determined by measuring ERK1/2 phosphorylation (**Figure 1**). Therefore, a reduction of 50% in ERK1/2 phosphorylation corresponds to 50% MEK1/2 inhibition. Nevertheless, cell-type-specific MEK-independent ERK phosphorylation has been reported in human neutrophil granulocytes (Simard et al., 2015). As the intended effect of an antiviral drug is a reduction in viral progeny, the EC_50_ value represents the zapnometinib concentration leading to a reduction of the virus titer by 50% (**Figure 1**). EC_50_ and IC_50_ are frequently used to compare the efficacy and potency of different drugs. However, a direct comparison can be difficult if these values were determined under different experimental conditions. Depending on a drug’s properties, such as binding to serum proteins, metabolization, temperature sensitivity of binding constants, or the timepoint they were assessed at, these values can change significantly (Caldwell et al., 2012; Pruijssers et al., 2020). For direct-acting antivirals, a direct correlation between the specific virus and the EC_50_ value can be expected. With zapnometinib targeting a host cell protein instead, the question arose, whether the EC_50_ value would be independent of the infecting virus under the same experimental conditions, as long as the infecting virus depends on an active Raf/MEK/ERK signaling pathway for its replication. Alternatively, different viruses may need different levels of MEK1/2 activity to sustain their replication and would therefore differ in their EC_50_ value. For development of a potential broad-spectrum antiviral, this information is especially important to predict the antiviral potential of the drug for other viruses and new arising virus species. In the present study, we therefore investigated if there is a difference in the dependency of SARS-CoV-2 and IAV on the Raf/MEK/ERK signaling pathway and subsequently on their susceptibility to zapnometinib treatment.

## 2 Methods

### 2.1 Viruses and cells

SARS-CoV-2 (B.1., Isolate “FI”, hCoV-19/Germany/FI1103201/2020) was taken from the strain repository of the Institute of Virology (IVM), Westfaelische Wilhelms-University Muenster, Germany (Schreiber et al., 2022), and passaged on Vero E6 cells (*Cercopithecus aethiops*, kidney epithelial cells) in a BSL3 laboratory. Influenza A virus, strain: A/Puerto Rico/8/1934 (H1N1) was obtained from Friedrich-Loeffler-Institute, Federal Research Centre for Virus Diseases of Animals, Tuebingen, Germany, and passaged on MDCK II cells in a BSL 2 laboratory. Calu-3 cells (*Homo sapiens* lung epithelial cells) and MDCK II cells (*Canis familiaris* kidney cells) were obtained from the American Type Culture Collection (ATCC). Caco-2 cells (*Homo sapiens* colon epithelial cells) were provided by the Institute for Medical Virology and Epidemiology of Viral Disease, Department of Molecular Virology, University Hospital Tuebingen, Germany. Cells were maintained in Iscove Modified Dulbecco Media (IMDM) (Thermo Fisher Scientific, Waltham, MA, USA) supplemented with 10% fetal bovine serum (FBS) (Capricorn Scientific GmbH, Ebsdorfergrund, Germany) and 1% penicillin–streptomycin (Sigma Aldrich, St. Louis, MO, USA) at 37°C, 5% CO_2_ in a humidified incubator.

### 2.2 Inhibitors

The MEK1/2 inhibitor Zapnometinib (PD184264, ATR-002), [2-(2-chloro-4-iodophenylamino)-N-3,4-difluoro benzoic acid], was synthesized at ChemCon, Freiburg, Germany, and provided by Atriva Therapeutics, Tuebingen, Germany.

### 2.3 Virus yield reduction assay

Calu-3 and Caco-2 cells were seeded in 24-well plates (Greiner Bio-One GmbH, Frickenhausen, Germany) and grown to 90% confluence. The cells were washed once with infection medium [IMDM supplemented with 0.2% bovine serum albumin (BSA) (Carl Roth GmbH + Co. KG, Karlsruhe, Germany) and 1% penicillin–streptomycin] prior to infection with a multiplicity of infection (MOI) of 0.01 of IAV PR8 or SARS-CoV-2 for 1 h in 200 µL of infection medium per well. Afterwards, the virus inoculum was removed completely, and the cells were washed once with infection medium and then treated with 1 mL per well of different concentrations of zapnometinib in infection medium with 1% dimethyl sulfoxide (DMSO) (Merck Millipore, Burlington, MA, USA) for 24 h at 37°C, 5% CO_2_. Supernatants were harvested and stored at −80°C for subsequent determination of virus titer and EC_50_ value. Cell lysates were prepared as described in the “Preparation of cell lysates and WES™ analysis” section for WES™ analysis.

### 2.4 Virus titer determination

The viral titers of IAV and SARS-CoV-2 viruses were determined using a real-time one-step multiplex RT-qPCR method. Briefly, viral nucleic acid extraction was performed using a QIAamp Viral RNA Mini Kit (Qiagen, Venlo, Netherlands) according to the manufacturer’s instructions. A TaqMan-based RT-qPCR assay was carried out using a QuantiNova Pathogen kit (Qiagen, Venlo, Netherlands) with specific primers targeting the SARS-CoV-2 N gene (2019-CoV2-N1F: AACACAAGCTTTCGGCAGAC, 2019-CoV2-N1R: ATTCCGAAGAACGCTGAAGC, 2019-CoV2-N1P [6FAM]: ACATTGGCCGCAAATTGCACAA [BHQ1]) and M gene for IAV according to the WHO guidelines. The viral genome copies per mL (gc/mL) were determined based on a standard curve prepared with 10-fold serial dilutions of the target gene. For quality control, nuclease-free water was included in each set of extractions as a negative control to monitor any possible cross-contamination. Moreover, an artificial exogenous QuantiNova RNA internal control (Qiagen, Venlo, Netherlands) was included during the nucleic acid purification to monitor the RNA purification efficiency and the RT-PCR amplification. A Rotor-Gene Q™ Real-Time PCR detection system (Qiagen, Venlo, Netherlands) was used for amplification, and the data were analyzed using Q-Rex software (Qiagen, Venlo, Netherlands).

### 2.5 EC_50_ determination

For EC_50_ determination, a virus yield reduction assay was performed. The virus titer in the cell culture supernatant was determined and normalized to the solvent control. The EC_50_ value was determined using the “log(inhibitor) vs. normalized response - Variable slope” equation in GraphPad Prism version 9.4.0.

### 2.6 Preparation of cell lysates and WES™ analysis

Cells were lysed using cold modified RIPA buffer [0.24% (w/v) tris base (Sigma-Aldrich, St. Louis, Missouri, USA), 0.88% (w/v) NaCl (Carl Roth, Karlsruhe, Germany), 0.2% (v/v) 500 mM ethylenediaminetetraacetic acid (EDTA) (Sigma-Aldrich, St. Louis, Missouri, USA), 1% (v/v) Triton X-100 (Sigma-Aldrich, St. Louis, Missouri, USA), 0.5% (w/v) sodium deoxycholate (Sigma-Aldrich, St. Louis, Missouri, USA), 0.1% (w/v) sodium dodecyl sulfate (SDS) (Carl Roth, Karlsruhe, Germany), 10% (v/v) glycerol (Carl Roth, Karlsruhe, Germany), 0.05% phenylmethylsulfonyl fluoride (PMSF) (Carl Roth, Karlsruhe, Germany), 0.01% Benzonase® Nuclease (Sigma-Aldrich, St. Louis, Missouri, USA), protease inhibitor cocktail (Roche, Basel, Switzerland), and phosphatase inhibitor cocktail (Roche, Basel, Switzerland) in water]. Determination of the protein concentration was performed using the Pierce™ BCA Protein Assay Kit (Thermo Fisher Scientific Inc., Waltham, MA, USA) according to the manufacturer’s instructions. The samples were analyzed by Wes™ using a total protein concentration of 0.25–0.5 µg/µL for each sample, the Jess/Wes Separation kit (12–230 kDa), primary antibodies against ERK1/2, p44/42 MAPK (137F5) Rabbit mAb (Cell Signaling Technology, Danvers, MA, USA, Cat. No. 4695S) and phospho-ERK1/2 (pERK1/2), Phospho-p44/42 MAPK (Erk1/2) (Thr202/Tyr204) (D13.14.4E), XP® Rabbit mAb (Cell Signaling Technology, Danvers, MA, USA, Cat. no. 4370S) in a 1:50 dilution, anti-Influenza A virus Nucleoprotein (NP) antibody (rabbit MAb) (Sino Biological, Beijing, China Cat.no 40208-R010) in a 1:150 dilution, SARS-CoV-2 nucleocapsid protein antibody (ProSci Inc., Poway, CA, USA, Cat. no. 35-580) in a 1:100 dilution, the anti-goat, anti-rabbit, and anti-mouse detection modules, and the EZ Standard Pack 1 (12–230 kDa), according to the manufacturer’s instructions. Analysis of the obtained data was performed using the Compass for SW 4.1.0 software, Microsoft Excel, and the GraphPad Prism 9.2.0 software. Ratios were calculated from the area under the curve of the respective detected signals.

### 2.7 IC_50_ determination

For determination of the IC_50_ value in the absence of viral infection, the cells were seeded in 24-well plates and incubated at 37°C, 5% CO_2_ until confluent. Twenty-four hours before the experiment, the growth medium was exchanged to medium with respective FBS or BSA concentration. Then, cells were stimulated by the addition of tumor necrosis factor alpha (TNF-α) (Sigma-Aldrich, St. Louis, Missouri, USA) for 30 min at 37°C, 5% CO_2_, followed by treatment with different zapnometinib concentrations and a DMSO (solvent) control for 1 h. For determination of the IC_50_ value in cells infected with IAV PR8 or SARS-CoV-2, cells were infected with the respective virus and treated with zapnometinib for 24 h as described in the “Virus yield reduction assay” section. After incubation, cell lysates were prepared, and the samples were analyzed by WES™ as described in the “Preparation of cell lysates and WES™ analysis” section. The pERK/ERK ratio was calculated and normalized to the solvent control. IC_50_ values were determined using the “log(inhibitor) vs. response -Variable slope (four parameters)” equation in GraphPad Prism version 9.4.0.

### 2.8 CC_50_ determination

Per well, 2.5 × 10^4^ Caco-2 cells or 4 × 10^4^ Calu-3 cells were seeded in 96-well plates (Greiner Bio-One, Kremsmünster, Austria) and incubated for 24 h at 37°C, 5% CO_2_. Cell viability was assessed using a WST-1 assay according to the manufacturer’s recommendations (Roche, Basel, Switzerland). For reference, cell viability was assessed at 0 h and after 24 h of treatment with different concentrations of zapnometinib in medium containing 1% DMSO. The measured cell viability was normalized to the 0-h control. The CC_50_ value was determined using the “Sigmoidal, 4PL, X is log(concentration)” equation in GraphPad Prism.

### 2.9 Flow cytometry analysis

For flow cytometry analysis, 6 × 10^5^ Caco-2 cells were seeded per well in six-well plates (Greiner Bio-One, Kremsmünster, Austria) and incubated for 24 h at 37°C, 5% CO_2_. Treatment with zapnometinib or a solvent control (1% DMSO) in medium containing 0.2% BSA was applied for 24 h. A single-cell solution was prepared using TrypLE reagent (Thermo Fisher Scientific, Waltham, MA, USA). Prior to staining, cells were treated with an FcR blocking reagent (Miltenyi Biotec, Bergisch Gladbach, Germany) for 10 min at 4°C. The cells were then stained with either a PE Mouse IgG1Isotype control antibody (BioLegend, San Diego, CA, USA) or a PE anti-human TMPRSS2 Antibody (BioLegend, San Diego, CA, USA), followed by a live-dead staining using the LIVE/DEAD™ Fixable Far Red Dead Cell Stain Kit (Thermo Fisher Scientific, Waltham, MA, USA). Cells were analyzed using the BD FACSCanto II flow cytometer (Becton Dickinson, Franklin Lakes, New Jersey, USA) and the FlowJo software (Becton Dickinson, Franklin Lakes, New Jersey, USA).

### 2.10 Immunofluorescence analysis

Caco-2 cells were seeded on glass cover slips in 24-well plates in IMDM medium containing 10% FBS and 1% penicillin/streptomycin and grown for 24 h at 37°C, 5% CO_2_. The next day, the cells were washed once with infection medium prior to infection with MOI 4 of SARS-CoV-2 or IAV PR8 for 1 h in infection medium per well. Afterwards, the virus inoculum was removed completely, and the cells were washed once with infection medium and treated with 1 mL per well with either different concentrations of zapnometinib or solvent control (1% DMSO) in infection medium for 10 h at 37°C, 5% CO_2_. Wells were washed with PBS and fixed with 4% paraformaldehyde (PFA) (Merck Millipore, Burlington, MA, USA) in PBS for 10 min at room temperature prior to permeabilization by the addition of 1 mL of 0.1% Triton X-100 (Merck Millipore, Burlington, MA, USA) in PBS per well for 5 min. Cells were blocked with 1% BSA in PBS for 1 h at room temperature before incubation with 200 µL of the primary antibody [Anti-SARS-CoV-2 nucleocapsid protein antibody (1:400) (ProSci Inc., Poway, CA, USA, Cat. no. 35-580) or Mouse anti-Influenza A virus Nucleoprotein (NP) antibody (1:500) (Bio-Rad Laboratories, Inc., Hercules, CA, USA, Cat No. MCA 400)] in 1% BSA in PBS overnight. The next day, cells were incubated with the secondary antibody [Alexa Fluor™ 488 goat anti-mouse IgG (1:1,000) (Thermo Fisher Scientific, Waltham, MA, USA)] in 1% BSA in PBS for 45 min at room temperature. Glass cover slips were removed from the wells and mounted on microscope slides using Roti^®^ mount FluorCare DAPI (Carl Roth GmbH + Co. KG, Karlsruhe, Germany). Slides were let dry at 4°C in the dark. Pictures were taken using a confocal laser scanning microscope with immersion oil and a 40× objective lens (LSM800, Zeiss, Oberkochen, Germany) and the Zen 3.3 software (Zeiss, Oberkochen, Germany).

## 3 Results

### 3.1 SARS-CoV-2 is susceptible to zapnometinib treatment

**Figure 2:**
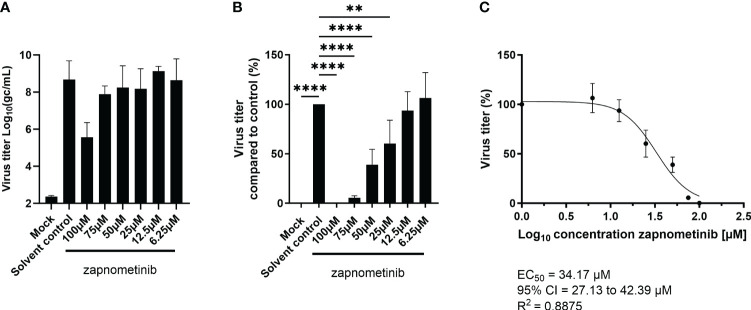
Determination of the EC_50_ value of zapnometinib in Caco-2 cells infected with MOI 0.01 of SARS-CoV-2. Zapnometinib treatment was initiated 1 h post-infection. The virus titer in the supernatant was determined after 24 h. **(A)** shows the measured virus titer in Log_10_(gc/mL), **(B)** the virus titer in percent compared to the solvent control and **(C)** the EC_50_ value determination of the data shown in **(B)**. Datapoints represent the means and SD of four independent experiments. Data passed a one-way ANOVA followed by Fisher’s LSD test [*p* > 0.05 (ns), *p* ≤ 0.01 (**), *p* ≤ 0.0001 (****)].

When the SARS-CoV-2 pandemic started in 2019, it was immediately tested if the replication of the virus was susceptible to treatment with the MEK1/2 inhibitor zapnometinib. Therefore, a virus yield reduction assay was performed on Caco-2 cells in medium containing 5% FBS. We found a concentration-dependent reduction of the virus titer for treatment with zapnometinib (**Figures 2A, B**). Compared to the solvent control, the virus titer was significantly reduced by zapnometinib treatment with 100 µM (reduction >99%), 75 µM (reduction >94%), 50 µM (reduction >60%), and 25 µM (reduction >39%). No significant reduction of the virus titer was achieved with treatment with 12.5 µM and 6.25 µM zapnometinib (**Figure 2B**). To calculate the EC_50_ value of zapnometinib, the measured virus titer in the different treatment conditions was normalized to the solvent control. The EC_50_ value of zapnometinib against SARS-CoV-2 was found to be 34.17 µM (**Figure 2C**), which is in line with the result obtained by other laboratories, e.g., 31.33 µM (SARS-CoV-2 B.1 isolate “FI”) by Schreiber and colleagues (Schreiber et al., 2022). In contrast to the previously determined EC_50_ values for IAV and influenza B virus (IBV) [6.36 µM (IAV RB1), 4.50 µM (IAV Fukui), and 4.19 µM (IBV Lee)] by Laure and colleagues (Laure et al., 2020), the EC_50_ value determined here for zapnometinib against SARS-CoV-2 is higher. We therefore wanted to investigate if this indicates that influenza virus is more susceptible to treatment with zapnometinib than SARS-CoV-2.

### 3.2 The IC_50_ value of zapnometinib is influenced by the serum concentration

**Figure 3:**
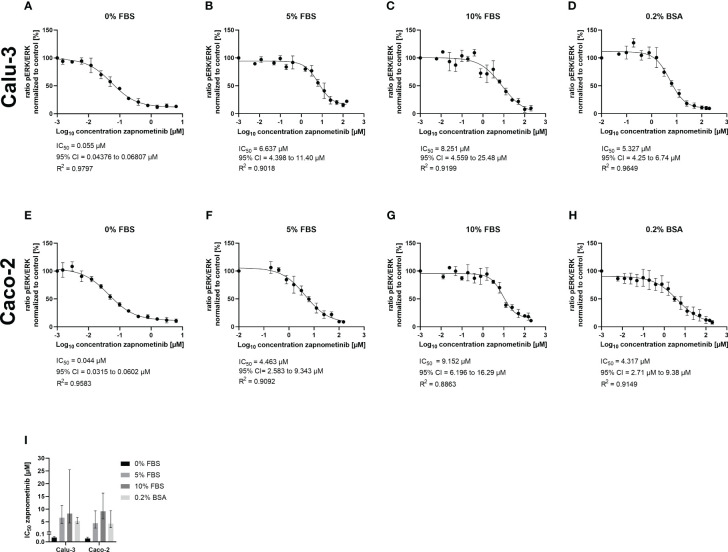
Development of the zapnometinib IC_50_ value in Caco-2 and Calu-3 cells in the presence of different concentrations of FBS or BSA during treatment. Caco-2 and Calu-3 cells were stimulated with TNF-α for 30 min followed by treatment with zapnometinib or a solvent control for an additional 1 h. Whole cell lysates were prepared and analyzed by WES™ for ERK1/2 and pERK1/2. The IC_50_ value determination of zapnometinib in Calu-3 cells for 0%, 5%, and 10% FBS and 0.2% BSA in the medium is shown in **(A–D)** and for Caco-2 cells in **(E–H)**. Datapoints represent the means and SD of independent experiments [**(A–G)** *n* = 3; **(H)** *n* = 4]. **(I)** Summarizes the IC_50_ values from **(A–H)**. Error bars represent the 95% confidence interval.

One factor that could be responsible for the different EC_50_ values is the plasma protein binding of the drug, which is a major issue in drug development. While a medium containing 5% FBS was used for SARS-CoV-2 infectivity assays (**Figure 2**), the EC_50_ values of zapnometinib against IAV and IBV were assessed using a medium containing 0.2% BSA instead (Laure et al., 2020). Thus, we aimed to investigate the influence of different FBS concentrations and 0.2% BSA in the absence of virus on the IC_50_ value of zapnometinib *in vitro* and thereby explore if the difference in the serum content of the media might have influenced zapnometinib’s efficacy in reducing ERK phosphorylation and thus led to the different EC_50_ values of zapnometinib seen for IAV and SARS-CoV-2. Furthermore, two different cell lines were tested to determine if the IC_50_ value may also be dependent on the cell line being used. Here, Calu-3 and Caco-2 cells were chosen, as both cell lines are susceptible to infection with SARS-CoV-2 as well as IAV PR8 and therefore provide a suitable basis for a direct comparison of these viruses. To determine the IC_50_ values, briefly the cells were stimulated with TNF-α for 30 min followed by treatment with zapnometinib or a solvent control. For both cell lines, we found a concentration-dependent increase of IC_50_ values for the different FBS concentrations with an IC_50_ value of 0.06 µM with 0% FBS, 6.64 µM with 5% FBS, and 8.25 µM with 10% FBS in the medium in Calu-3 cells. In Caco-2 cells, the IC_50_ values were in a similar range, with 0.04 µM with 0% FBS, 4.46 µM with 5% FBS, and 9.15 µM with a content of 10% FBS in the medium. Medium containing 0.2% BSA resulted in an IC_50_ of 5.33 µM in Calu-3 and 4.32 µM in Caco-2 cells (**Figure 3**). The present data demonstrate that the FBS concentration in the medium has a strong effect on the IC_50_ of zapnometinib. Overall, the determined IC_50_ values were similar for both cell lines in each condition. We therefore conclude that Caco-2 and Calu-3 cells respond to treatment with zapnometinib in a similar manner.

### 3.3 The EC_50_ value of zapnometinib depends on the infecting virus

**Figure 4:**
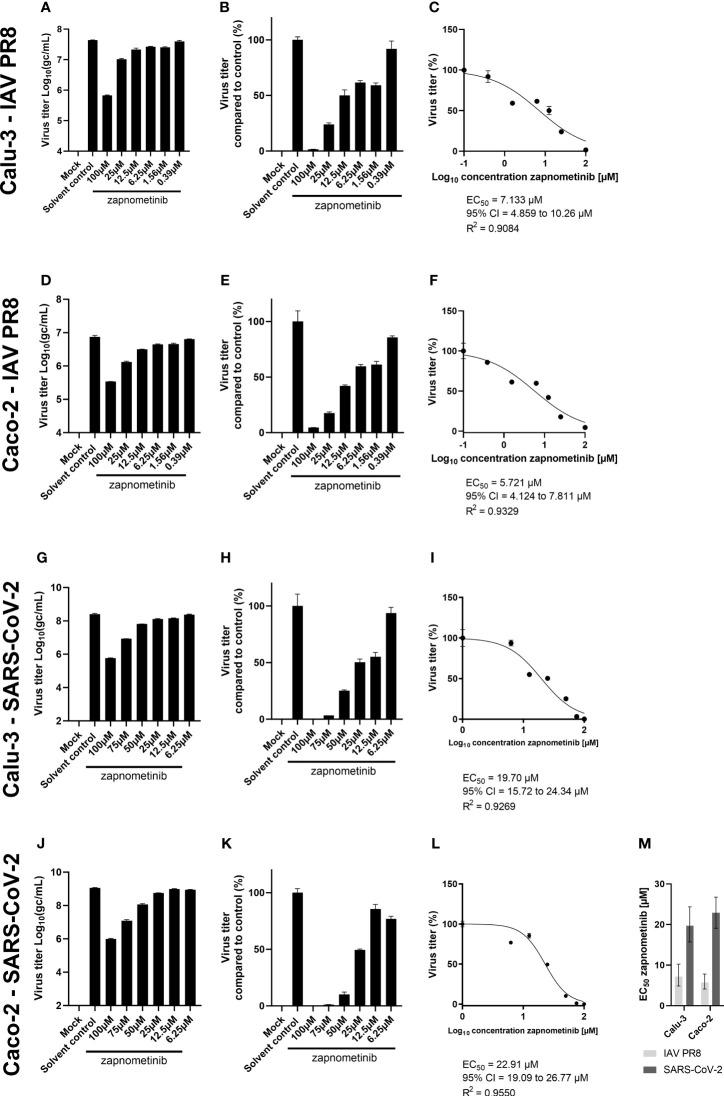
Determination of the EC_50_ value of zapnometinib against SARS-CoV-2 and IAV PR8 in Caco-2 and Calu-3 cells. Zapnometinib treatment was initiated 1 h post-infection. The virus titer in the supernatant was determined after 24 h. The respective cell line and virus are indicated on the left side of each row of graphs. Datapoints represent the means and SD of pooled biological triplicates measured in technical triplicates. **(A, D, G, J)** Show the measured virus titer in Log_10_(gc/mL). **(B, E, H, K)** Show the virus titer in percent compared to the solvent control. **(C, F, I, L)** Show the EC_50_ value determination of the data presented in **(B, E, H, K)**. **(M)** summarizes the EC_50_ values from **(C, F, I, L)**. Error bars represent the 95% confidence interval.

Next, to exclude the potential influence of different media compositions, we tested if infection of Caco-2 and Calu-3 cells with SARS-CoV-2 using cell culture medium containing 0.2% BSA, which is routinely used for IAV infections, would also support successful replication of SARS-CoV-2. We found that SARS-CoV-2 replication was not affected by the medium change, and virus titers of approximately 1 × 10^9^ gc/mL, as observed in medium containing 5% FBS, were achieved after 24 h (see SARS-CoV-2 solvent control, **Figures 2**, **4**). These results allowed the conduct of EC_50_ experiments with IAV PR8 and SARS-CoV-2 using the same experimental conditions, meaning the same cell lines and medium composition. We performed virus yield reduction assays on Caco-2 and Calu-3 cells using IMDM medium containing 0.2% BSA. Briefly, cells were infected with MOI 0.01 of IAV PR8 or SARS-CoV-2 followed by treatment with different zapnometinib concentrations of up to 100 µM for 24 h. The concentrations used have been shown to cause no cytotoxic effects on Calu-3 and Caco-2 cells (**Supplementary Figure 1**). After 24 h of treatment, the virus titer was determined in the supernatant (**Figures 4A, D, G, J**). For both viruses, we found a concentration-dependent reduction of the virus titer under zapnometinib treatment compared to the solvent control on both cell lines. The virus titers were normalized to the respective solvent controls (**Figures 4B, E, H, K**), followed by the determination of the respective EC_50_ values (**Figures 4C, F, I, L**). For IAV PR8, we found a reduction of the virus titer of >90% under treatment with 100 µM and a reduction of the virus titer of ≥50% for treatment with 25 µM and 12.5 µM zapnometinib. For SARS-CoV-2, the reduction of the virus titer was >90% for treatment with 100 µM and 75 µM zapnometinib and ≥50% for treatment with 50 µM and 25 µM zapnometinib. For IAV PR8, the determined EC_50_ value was 7.13 µM in Calu-3 cells and 5.72 µM on Caco-2 cells (**Figures 4C, F**). The EC_50_ values determined for SARS-CoV-2 (FI) were 19.70 µM on Calu-3 cells and 22.91 µM on Caco-2 cells (**Figures 4I, L**). EC_50_ values below 10 µM for IAV PR8, and EC_50_ values of approximately 20 µM for SARS-CoV-2 in both cell lines, clearly demonstrate that IAV and SARS-CoV-2 differ in their dependency on an active Raf/MEK/ERK signaling pathway.

### 3.4 The amount of SARS-CoV-2 N and IAV NP is reduced in infected cells under zapnometinib treatment

**Figure 5:**
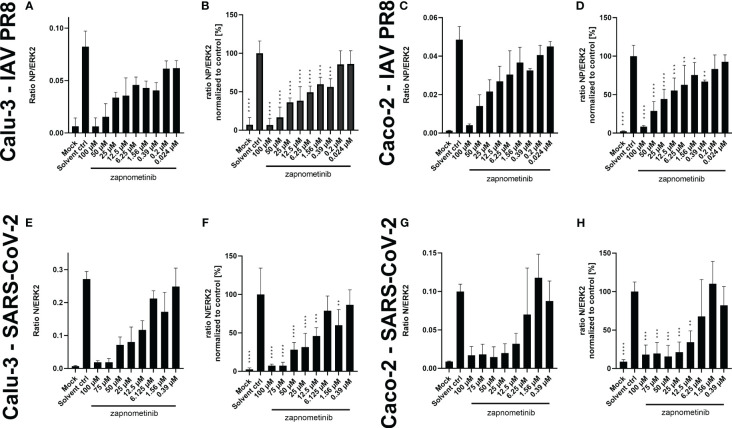
Amount of SARS-CoV-2 N and IAV PR8 NP normalized to ERK2 in whole cell lysates of Caco-2 and Calu-3 cells infected with MOI 0.01 of SARS-CoV-2 and IAV PR8 and treated with zapnometinib or a solvent control. Treatment was initiated 1 h post-infection. Whole cell lysates were prepared after 24 h and analyzed by WES™ for the SARS-CoV-2 N and IAV NP and ERK2. The respective cell line and virus are indicated on the left side of each row of graphs. Datapoints represent the mean and SD of ≥3 biological replicates. **(A, C)** Show the ratio of the IAV NP to ERK2 as reference protein and **(E, G)** show the ratio of the SARS-CoV-2 N to ERK2 as reference protein. **(B, D, F, H)** Show the (N/NP)/ERK2 ratio normalized to the solvent control of the respective experiments. Data passed a one-way ANOVA followed by Fisher’s LSD test [*p* > 0.05 (ns), *p* ≤ 0.05 (*), *p* ≤ 0.01 (**), *p* ≤ 0.001 (***), *p* ≤ 0.0001 (****)].

Furthermore, we investigated if the reduction seen in the virus titer under treatment with zapnometinib is also reflected in the amount of viral protein in the cells. Therefore, we analyzed the SARS-CoV-2 nucleocapsid protein (N) and IAV nucleoprotein (NP) content in the lysates of infected Caco-2 and Calu-3 cells after 24 h of zapnometinib treatment. The detected amount of both proteins was related to ERK2 as reference protein (**Figures 5A, C, E, G**) and normalized to the solvent control (**Figures 5B, D, F, H**). Zapnometinib treatment does not affect ERK2 content in the cells (**Supplementary Figure 4**). In agreement with the reduction seen for the virus titer, we found a concentration-dependent reduction of the SARS-CoV-2 N and IAV PR8 NP under zapnometinib treatment in both cell lines by >80% with 100 µM and >70% with 50 µM zapnometinib, respectively. A reduction of approximately 50% was reached under treatment with 12.5 µM zapnometinib. A difference in the reduction of the SARS-CoV-2 N and IAV NP was observed at lower concentrations. The IAV PR8 NP was significantly reduced compared to the solvent control under treatment with up to 0.39 µM zapnometinib while the SARS-CoV-2 N was significantly reduced only under treatment with up to 12.5 µM zapnometinib (**Figures 5B, D, F, H**). The fact that the IAV NP is still reduced at lower concentrations of zapnometinib further supports the results from the EC_50_ experiment that IAV is more susceptible to zapnometinib treatment than SARS-CoV-2.

### 3.5 IC_50_ value of zapnometinib in SARS-CoV-2 or IAV PR8-infected Caco-2 and Calu-3 cells

**Figure 6:**
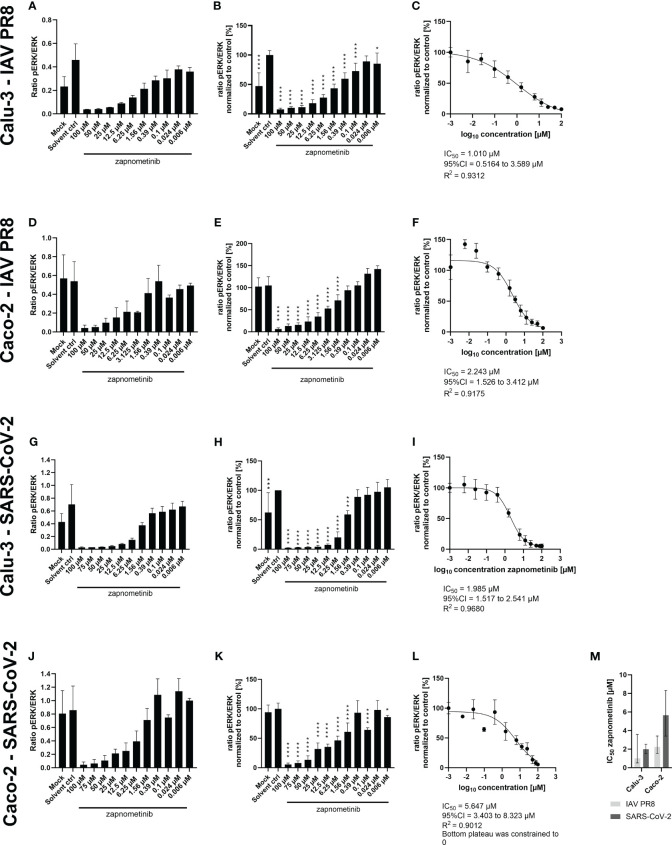
Determination of the IC_50_ value of zapnometinib in Caco-2 and Calu-3 cells infected with MOI 0.01 of SARS-CoV-2 and IAV PR8 and treated with zapnometinib. Zapnometinib treatment was initiated 1 h post-infection. Whole cell lysates were prepared after 24 h and analyzed by WES™ for ERK1/2 and pERK1/2. The respective cell line and virus are indicated on the left side of each row of graphs. Datapoints represent the means and SD of three biological replicates. **(A, D, G, J)** Show the ratio of pERK1/2 to ERK1/2. **(B, E, H, K)** Show the pERK1/2/ERK1/2 ratio normalized to the solvent control of the respective experiments. Data passed a one-way ANOVA followed by Fisher’s LSD test [*p* > 0.05 (ns), *p* ≤ 0.05 (*), *p* ≤ 0.01 (**), *p* ≤ 0.001 (***), *p* ≤ 0.0001 (****)]. **(C, F, I, L)** Show the IC_50_ value determination of the data shown in **(B, E, H, K)**. **(M)** summarizes the IC_50_ values from **(C, F, I, L)**. Error bars represent the 95% confidence interval.

Next, we determined the IC_50_ value of zapnometinib in virus-infected cells to investigate the activation level of the Raf/MEK/ERK signaling pathway in infected cells under treatment with zapnometinib. Therefore, Caco-2 and Calu-3 cells were infected for 1 h with MOI 0.01 of IAV PR8 or SARS-CoV-2, respectively, followed by treatment with different concentrations of zapnometinib for 24 h. The ratio of pERK1/2 to ERK1/2 was calculated (**Figures 6A, D, G, J**) and normalized to the solvent control to determine the IC_50_ value (**Figures 6B, E, H, K**). Compared to the non-infected mock control, we found an activation of the Raf/MEK/ERK pathway in IAV PR8-infected Calu-3 cells with an increase in the pERK/ERK ratio by 0.23 in the solvent control (**Figure 6A** and **Table 1**). Normalized to the solvent control, this results in an activation by 52% (**Figure 6B**). In SARS-CoV-2-infected Calu-3 cells, the pERK/ERK ratio increased by 0.28 in the solvent control compared to mock, which translates into an activation by 31% (**Figures 6G, H** and **Table 1**). Interestingly, we did not observe an increase in ERK1/2 phosphorylation in the solvent control of infected Caco-2 cells compared to mock. Looking at the level of ERK phosphorylation in the Caco-2 mock control, the MEK activity was already at a level that was reached in Calu-3 cells only after virus infection (ratio pERK/ERK Caco-2 IAV-PR8 mock: 0.55, Calu-3 IAV PR8 solvent ctrl: 0.45, Caco-2 SARS-CoV-2 mock: 0.81, Calu-3 SARS-CoV-2 solvent ctrl: 0.7) (**Figure 6** and **Table 1**). This preactivation of the pathway might explain why there is no further increase in ERK phosphorylation upon infection. Zapnometinib treatment led to a concentration-dependent reduction in ERK1/2 phosphorylation in all set ups. The IC_50_ value of zapnometinib was 1.01 µM in IAV PR8-infected Calu-3 cells and 2.24 µM in Caco-2 cells. In SARS-CoV-2-infected cells, the IC_50_ value was 1.99 µM in Calu-3 and 5.65 µM in Caco-2 cells (**Figures 6C, F, I, L, M**). In **Figure 6M**, the IC_50_ values are summarized. For both viruses, the IC_50_ value of zapnometinib was lower in Calu-3 cells compared to Caco-2 cells, indicating that lower amounts of zapnometinib are needed to achieve 50% MEK inhibition in virus-infected Calu-3 cells. Furthermore, the IC_50_ value in IAV PR8-infected cells was lower compared to SARS-CoV-2-infected cells, demonstrating that 50% MEK inhibition is reached at lower concentrations in IAV PR8-infected cells, which might explain why the EC_50_ value of zapnometinib against IAV PR8 is lower compared to the EC_50_ value against SARS-CoV-2. Curve fitting to determine the IC_50_ values allowed us to interpolate the level of MEK activation at the respective EC_50_ values determined in **Figure 4**. The MEK activity at the EC_50_ concentration of zapnometinib against IAV PR8 and SARS-CoV-2 was 25% and 10% in Calu-2 cells and 37% and 25% in Caco-2 cells, respectively (**Table 2**). These results show that higher levels of MEK inhibition are needed to reduce the virus titer by 50% for SARS-CoV-2 compared to IAV in both cell lines.

**Table 1:** Ratio pERK/ERK in virus-infected Calu-3 and Caco-2 cells (see also **Figure 6**).

Cell line	Virus	Mock Ratio pERK/ERK [Mean, SD]	Solvent control Ratio pERK/ERK [Mean, SD]
**Calu-3**	**IAV PR8**	0.23 ± 0.08	0.45 ± 0.13
	**SARS-CoV-2**	0.43 ± 0.13	0.7 ± 0.28
**Caco-2**	**IAV PR8**	0.55 ± 0.23	0.55 ± 0.25
	**SARS-CoV-2**	0.81 ± 0.33	0.86 ± 0.33

**Table 2:** Interpolated MEK activity at the EC_50_ value of zapnometinib in IAV PR8 and SARS-CoV-2-infected Calu-3 and Caco-2 cells.

Cell line	Virus	EC_50_ [µM]	Level of MEK activation (ERK phosphorylation) at EC_50_ [%]
**Calu-3**	IAV PR8	7.13	25%
	SARS-CoV-2	19.7	10%
**Caco-2**	IAV PR8	5.72	37%
	SARS-CoV-2	22.9	25%

### 3.6 Influence of MEK inhibition on virus entry and the N/NP localization

**Figure 7:**
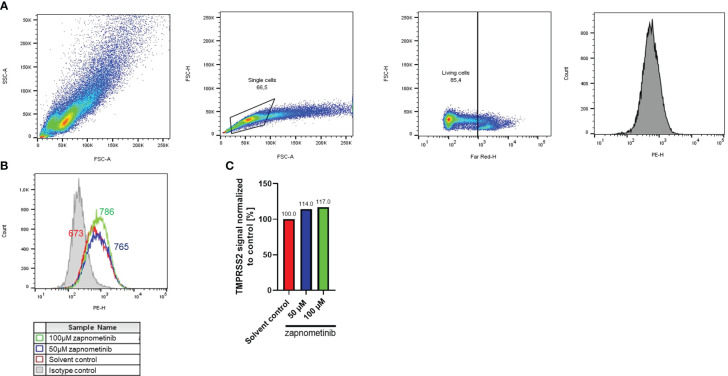
TMPRSS2 expression after zapnometinib treatment in Caco-2 cells. Caco-2 cells were treated with zapnometinib or a solvent control for 24 h followed by staining for TMPRSS2 (PE) and Live/Dead cells (Far-Red). Cells were analyzed by flow cytometry and gated for single, living cells prior to detection of TMPRSS2. Shown is the gating strategy of a representative sample **(A)**, the TMPRSS2 signal **(B)**, and the median TMPRSS2 signal of each sample normalized to the solvent control **(C)**.

SARS-CoV-2 and IAV can use TMPRSS2 to facilitate entry. Thus, we investigated if MEK inhibition influences TMPRSS2 expression as a potential common mechanism of action for the antiviral activity against SARS-CoV-2 and IAV PR8. Therefore, Caco-2 cells were analyzed by flow cytometry for their TMPRSS2 expression with either zapnometinib or a solvent control. Surprisingly, the TMPRSS2 expression in Caco-2 cells increased by 14% and 17% under treatment with 50 µM and 100 µM zapnometinib, respectively (**Figure 7**).

**Figure 8:**
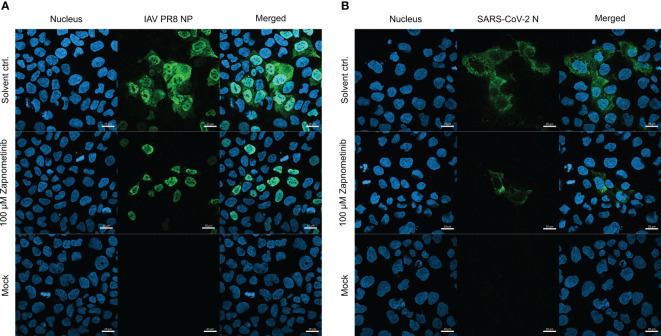
Localization of the IAV PR8 NP and SARS-CoV-2 N under treatment with zapnometinib compared to a solvent control. Caco-2 cells were infected with MOI 4 of either IAV PR8 **(A)** or SARS-CoV-2 **(B)** for 1 h. Mock-infected cells received infection medium. The inoculum was removed, and the cells were incubated for 10 h with either DMSO (solvent control) or 100 µM zapnometinib, followed by immunofluorescence staining for the viral nucleoprotein (green). Nuclei were stained with 4′,6-diamidino-2-phenylindole (blue). The pictures were taken with a 40× objective lens with immersion oil using an LSM 800 microscope (Zeiss, Oberkochen, Germany). Scale bars indicate 20 µm.

To visualize the different localization of the SARS-CoV-2 N and IAV NP, an immunofluorescence analysis was performed. Caco-2 cells were either infected with IAV PR8 or SARS-CoV-2 for 1 h followed by treatment with 100 µM zapnometinib or a solvent control for 10 h. The cells were fixed and stained for the nucleus and the respective viral protein. In the solvent control of the IAV PR8-infected Caco-2 cells (**Figure 8A**), the NP is distributed throughout the whole cell (nucleus and cytoplasm), while the SARS-CoV-2 NP (**Figure 8B**) is located exclusively in the cytoplasm; the nuclear area is clear. Treatment with zapnometinib led to the retention of the IAV NP in the nucleus, as observed earlier with other MEK inhibitors (Pleschka et al., 2001; Schreiber et al., 2020). In contrast, the distribution of the SARS-CoV-2 N did not change. However, the amount of SARS-CoV-2 N was reduced, and fewer SARS-CoV-2 N-positive cells were detected, compared to IAV PR8 NP-positive cells post-treatment (**Figure 8**; **Supplementary Figure 3**).

## 4 Discussion

Viruses depend on their host cell’s machinery to enable replication. Therefore, targeting host cell proteins to prevent viral replication is a promising strategy to develop long-lasting broad-spectrum antiviral drugs. The small-molecule MEK inhibitor zapnometinib blocks the signal transduction of the Raf/MEK/ERK signaling pathway, a pathway hijacked by numerous viruses during their replication cycle. It is tempting to speculate that inhibition of that pathway to a specific extent would prevent propagation of all viruses that depend on it. When first investigating the EC_50_ value of zapnometinib against SARS-CoV-2 in early 2020, surprisingly the EC_50_ value was higher compared to previously determined EC_50_ values against IAV and IBV. To scrutinize if this was a secondary effect due to different experimental conditions or if the replication cycle of SARS-CoV-2 was less dependent on the Raf/MEK/ERK signaling pathway, we searched for conditions that would enable the EC_50_ determination for both viruses in a comparable manner. Interestingly, under similar experimental conditions, the EC_50_ values were still lower for IAV, suggesting that IAV is more susceptible to zapnometinib treatment (**Figure 4**). Thus, it seems that IAV has a higher dependency on an active Raf/MEK/ERK signaling pathway compared to SARS-CoV-2. In line with this hypothesis, we found a stronger activation of the Raf/MEK/ERK signaling pathway in IAV-infected Calu-3 cells (52%) compared to SARS-CoV-2-infected cells (30%) compared to the respective non-infected mock control (**Figures 6B, H**). The extent to which the viruses activate the Raf/MEK/ERK signaling pathway could only be assessed in Calu-3 cells, as the pERK/ERK ratio in Caco-2 cells was the same in non-infected and infected cells (**Figures 6E, K**). Furthermore, the pERK/ERK ratio in the mock controls in the SARS-CoV-2 experiments was higher compared to IAV experiments (**Table 1**). We focused on the normalized data for the direct comparison to compensate for this effect.

The EC_50_ values clearly demonstrate that less zapnometinib is needed to reduce IAV virus titer by 50% compared to SARS-CoV-2, but so far, the level of MEK inhibition at this concentration remained unknown. The IC_50_ experiments in virus-infected cells performed here completed the picture and allowed a better understanding of the level of MEK activity during infection and treatment. Interpolation of the level of MEK activity from the IC_50_ data in virus-infected cells at the EC_50_ value of zapnometinib revealed that the lower EC_50_ values determined for IAV PR8 compared to SARS-CoV-2 also translate into lower levels of MEK inhibition compared to SARS-CoV-2, meaning less MEK inhibition is needed to reduce the virus titer of IAV PR8 by 50% compared to SARS-CoV-2 (**Table 1**).

Many viruses depend on proteolytic cleavage of surface proteins by host cell proteases like, e.g., furin, cathepsins, or TMPRSS2 to facilitate their entry. The serine protease TMPRSS2 plays a role during the entry of both IAV and SARS-CoV-2, and its expression has been described to be regulated via the androgen receptor (Böttcher-Friebertshäuser et al., 2010; Hoffmann et al., 2020; Baratchian et al., 2021), which, in turn, has been shown to be regulated by the Raf/MEK/ERK signaling pathway (Yeh et al., 1999; Carey et al., 2007). As a potential common mode of action for zapnometinib in inhibiting both viruses, we investigated if TMPRSS2 expression was influenced by treatment with zapnometinib. Although we found a slight increase in the expression of TMPRSS2 in Caco-2 cells, which, seen by itself, could be considered adverse, zapnometinib treatment still has a strong antiviral effect against both viruses. Therefore, we consider the observed increase in TMPRSS2 expression post-treatment negligible. Surprisingly, we were not able to detect TMPRSS2 expression in Calu-3 cells (**Supplementary Figure 2**) although Calu-3 cells are known to express TMPRSS2 (Chen et al., 2021; Yu et al., 2022). Further studies need to be conducted to investigate the mode of action of zapnometinib during entry.

IAV and SARS-CoV-2 both present with an early activation of the Raf/MEK/ERK pathway upon infection. For SARS-CoV-2, it is known that binding of the spike protein to the cellular ACE2 receptor directly leads to an activation of the Raf/MEK/ERK signaling pathway (Suzuki et al., 2021). Similar events have been described for IAV; e.g., interaction of the virus with the platelet-derived growth factor receptor (PDGFRβ) during entry led to the activation of the Raf/MEK/ERK signaling pathway (Vrijens et al., 2019). The downstream processes induced by this early activation are still unknown for SARS-CoV-2. However, the reduced number of N-positive cells in the immunofluorescence analysis under zapnometinib treatment shows that MEK inhibition interferes with SARS-CoV-2 entry. For influenza virus on the other hand, Marjuki et al. could show that the early activation of the Raf/MEK/ERK pathway led to an increase in V-ATPase activity, which, in turn, led to endosomal acidification necessary for the fusion of the virus particle (Marjuki et al., 2011). In support of the involvement of the Raf/MEK/ERK pathway during entry, we found the number of IAV NP-positive cells to be reduced in the immunofluorescence analysis, while not as strongly as for SARS-CoV-2 N (**Supplementary Figure 3**).

The late activation of the Raf/MEK/ERK pathway differentiates IAV from SARS-CoV-2 and provides a potential explanation for the increased susceptibility of IAV towards zapnometinib treatment. It is triggered by accumulation of the IAV HA at the cell membrane and has been shown to be required for the successful export of the ribonucleoprotein complexes from the nucleus to the cell membrane (Marjuki et al., 2006; Schreiber et al., 2020). Thus, while SARS-CoV-2 activates the Raf/MEK/ERK signaling pathway only once during entry, IAV is dependent on an active Raf/MEK/ERK signaling pathway during two crucial stages of its replication cycle: entry and the export of the ribonucleoprotein complexes from the nucleus, which may lead to its higher sensitivity to MEK inhibition.

In conclusion, we could show that the EC_50_ and IC_50_ value of zapnometinib against IAV are lower compared to SARS-CoV-2 in Calu-3 and Caco-2 cells and that the level of MEK inhibition at the EC_50_ value of zapnometinib against IAV is lower compared to SARS-CoV-2. Therefore, we conclude that IAV’s replication has a stronger dependency on an active Raf/MEK/ERK signaling pathway than SARS-CoV-2, and following that, IAV is more susceptible to zapnometinib. Despite SARS-CoV-2 being less sensitive to zapnometinib *in vitro*, the recently conducted human phase II clinical trial in hospitalized coronavirus disease 2019 (COVID-19) patients has indicated a clinically relevant efficacy profile for zapnometinib, in terms of the primary endpoint, with a favorable safety profile (ClinicalTrials.gov NCT04776044). In the light of the present study, these results are promising for an upcoming phase II clinical trial for treatment of severe influenza virus infection and pandemic preparedness in general.

## Data availability statement

The original contributions presented in the study are included in the article/**Supplementary Material**. Further inquiries can be directed to the corresponding author.

## Ethics statement

Ethical approval was not required for the studies on humans in accordance with the local legislation and institutional requirements because only commercially available established cell lines were used.

## Author contributions

HHo: Conceptualization, Investigation, Methodology, Writing – original draft, Writing – review & editing, Project administration. ME: Investigation, Writing – review & editing, Methodology. ASS: Investigation, Writing – review & editing. HHa: Investigation, Writing – review & editing. JK: Writing – review & editing, Investigation. ASB: Writing – review & editing. SL: Writing – review & editing. MS: Resources, Writing – review & editing. OP: Writing – review & editing, Conceptualization, Resources, Supervision.
