# Supplementary material for: Influenza A virus replication has a stronger dependency on Raf/MEK/ERK signaling pathway activity than SARS-CoV-2
Source: Front Cell Infect Microbiol. 2023 Oct 26;13:1264983. doi: 10.3389/fcimb.2023.1264983 (PMC10641236; doi:10.3389/fcimb.2023.1264983)
Supplement: Supplementary file 1 [file DataSheet_1.pdf]

Supplementary Material

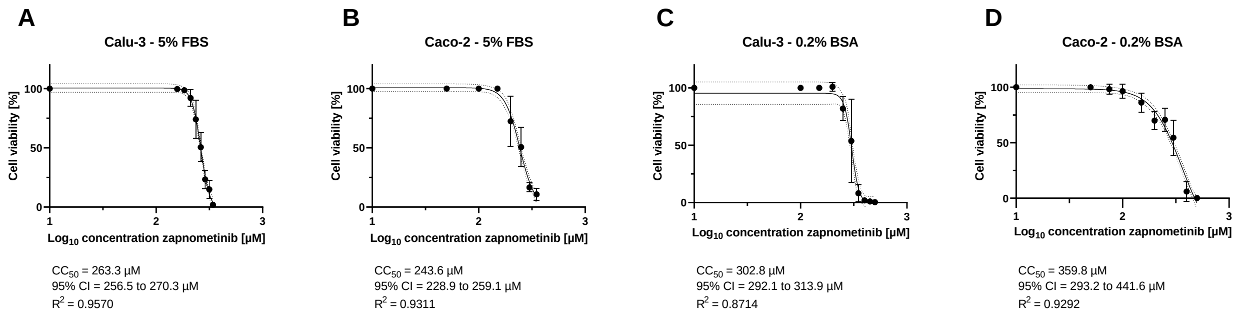

Supplementary Figure 1: CC_{50} value of zapnometinib in Caco-2 and Calu-3 cells in medium containing 5% FBS or 0.2% BSA. Caco-2 and Calu-3 cells were treated with different concentrations of zapnometinib for 24h. The cell viability was assessed and normalized to a 0h control. The CC_{50} value determination of zapnometinib in Calu-3 and Caco-2 cells for medium containing 5% FBS is shown in (A) and (B) and for medium containing 0.2% BSA in (C) and (D). Datapoints represent the means and SD of three independent experiments.

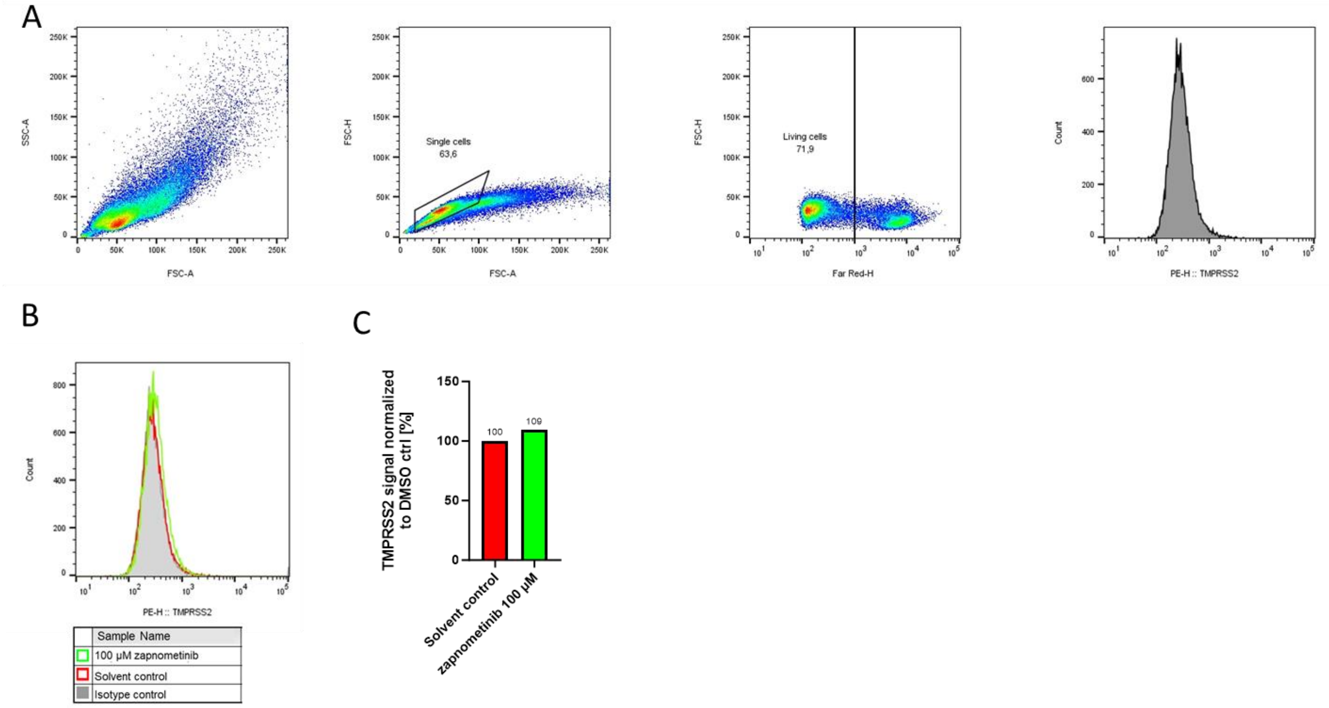

Supplementary Figure 2: TMPRSS2 expression after zapnometinib treatment in Calu-3 cells. Calu-3 cells were treated with zapnometinib or a solvent control for 24h followed by staining for TMPRSS2 (PE) and Live/Dead cells (Far-Red).
Cells were analyzed by flow cytometry and gated for single, living cells prior to detection of TMPRSS2. Shown is the gating strategy of a representative sample (A), the TMPRSS2 signal (B) and the TMPRSS2 signal normalized to the solvent control (C).

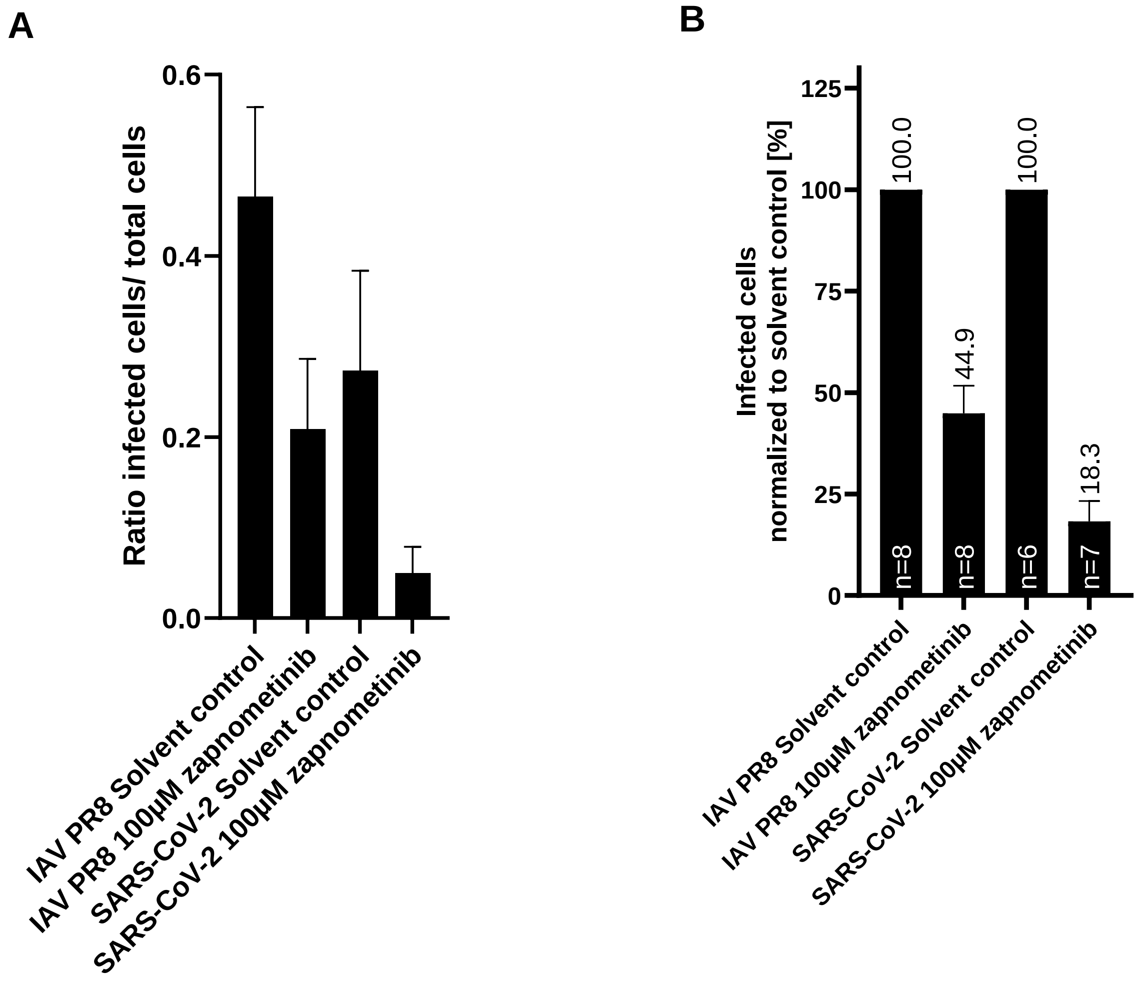

Supplementary Figure 3: Reduced number of IAV PR8 NP and SARS-CoV-2 N positive cells post zapnometinib treatment. Caco-2 cells were infected with MOI 4 of either IAV PR8 or SARS-CoV-2 for 1h. The inoculum was removed, and the cells were incubated for 10h with either DMSO (solvent control) or 100µM zapnometinib, followed by immunofluorescence staining for the viral nucleoprotein. Nuclei were stained with 4′,6-diamidino-2-phenylindole. Pictures were taken with a 40x objective lens with immersion oil using a LSM 800 microscope (Zeiss, Oberkochen, Germany). For each picture IAV PR8 NP / SARS-CoV-2 N positive cells and the total amount of nuclei were counted. (A) shows the ratio of IAV PR8 NP / SARS-CoV-2 N positive cells to the total cell count (nuclei). (B) shows the amount of IAV PR8 NP and SARS-CoV-2 N positive cells in percent, normalized to the respective solvent control (n= number of pictures analyzed). It is important to emphasize that this evaluation is not free of a bias, especially with the low number of infected cells for SARS-CoV-2 after treatment. As pictures were taken that show at least one infected cell for evaluation of the localization of the IAV PR8 NP and SARS-CoV-2 N, as shown in Figure 8.

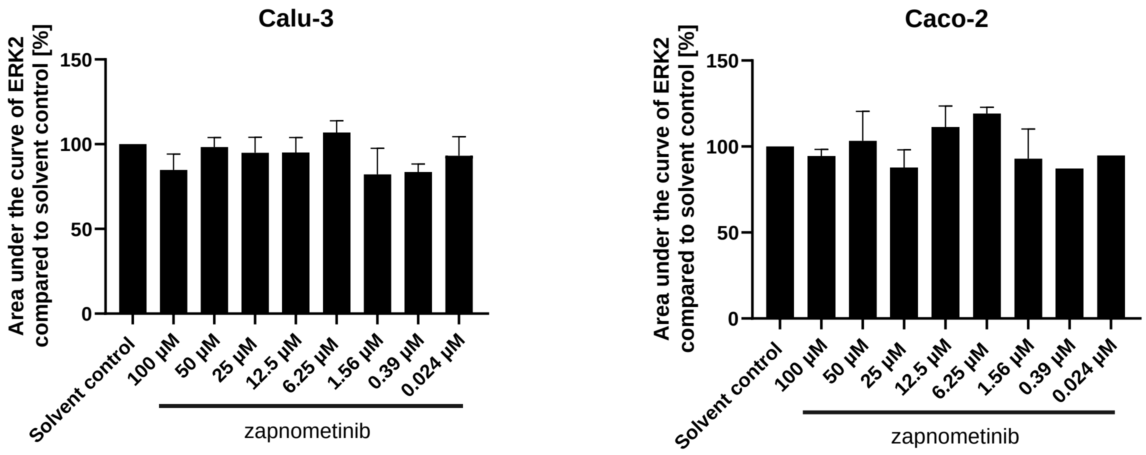

Supplementary Figure 4: Amount of ERK2 in cell lysates under treatment with zapnometinib. Caco-2 and Calu-3 cell lysates analysed by Wes™ for ERK2 after 24h of treatment with different zapnometinib concentrations (n=3).
